# Identification of CKS1B as a prognostic indicator and a predictive marker for immunotherapy in pancreatic cancer

**DOI:** 10.3389/fimmu.2022.1052768

**Published:** 2022-11-03

**Authors:** Lincheng Li, Jing Wang, Zhuochao Zhang, Qiyue Yang, Zhaoda Deng, Wenbo Zou, Xinlan Ge, Ke Pan, Chonghui Li, Rong Liu

**Affiliations:** ^1^ Medical School of Chinese PLA, Beijing, China; ^2^ Faculty of Hepato-Pancreato-Biliary Surgery, The First Medical Center, Chinese PLA General Hospital, Beijing, China; ^3^ Key Laboratory of Digital Hepetobiliary Surgery, PLA, Institute of Hepatobiliary Surgery of Chinese PLA, Beijing, China; ^4^ Department of General Surgery, No.924 Hospital of PLA Joint Logistic Support Force, Guilin, China

**Keywords:** CKS1B, prognosis, PD-L1, pancreatic cancer, immunotherapy

## Abstract

As a regulatory subunit of cyclin kinase, CKS1B promotes cancer development and is associated with poor prognosis in multiple cancer patients. However, the intrinsic role of CKS1B in pancreatic cancer remains elusive. In our research, CKS1B expression in pancreatic tumor tissue was higher than that in normal tissue by TCGA, Oncomine and CPTAC databases analysis. Similar result was verified in our center tissues by qRT-PCR. CKS1B expression was closely relevant to histologic grading, prognosis, and TMB. GSEA showed that CKS1B mainly participated in the regulation of autophagy and T cell receptor signaling pathway. Furthermore, CIBERSORT analysis showed that there was a strong correlation between CKS1B expression and tumor immune cells infiltration. Drug sensitivity analysis showed that patients with high CKS1B expression appeared to be more sensitive to gemcitabine, 5-fluorouracil, and paclitaxel. We then investigated cell viability and migratory ability by CCK8 and transwell assay, respectively. Results indicated that CKS1B knockdown by short hairpin RNA significantly reduced pancreatic cancer cell viability and invasion *via* regulating PD-L1 expression. In conclusion, our research further demonstrates the role of CKS1B in pancreatic cancer and the signaling pathways involved. The association of CKS1B with immune infiltration and immune checkpoint may provide a new direction for immunotherapy of pancreatic cancer.

## Introduction

Pancreatic cancer is among the deadliest malignancies with unfavorable diagnostic accuracy and patients’ prognoses. Due to the aggressive nature of early pancreatic cancer cells, it is difficult to completely cure pancreatic cancer through surgery (1). Despite advances in existing treatments for pancreatic cancer including surgery, immunotherapy, and chemotherapy, the patients’ five-year survival rate is below 10% worldwide (2). Hence, it is of great significance to conduct research on the underlying mechanism of pancreatic cancer and to discover novel therapeutic targets for the disease.

Malignant tumors result from cell cycle dysfunction and aberrant cell differentiation. CDC28 protein kinase regulatory subunit 1B (CKS1B), which belongs to the CKS family, participates in the modulation of cell cycle function by binding to the CDK’s catalytic subunit (3). The initiation and development of many malignant tumors are related to the overexpression of CKS1B, such as colon cancer, lung cancer, gastric cancer, and breast cancer (4–6). Deng et al. found that CKS1B silencing inhibites cell proliferation and invasion and activates apoptosis in glioma (7). In addition, a meta-analysis including 2,224 cancer participants showed that high CKS1B expression is associated with advanced T stage and lymph node metastasis (8). These evidences suggest that CKS1B may be a key gene to promote the malignant progression in a variety of tumors. Furthermore, CKS1B has been identified as a ubiquitin-like protein system resistance gene that can induce resistance to inhibitors of ubiquitin-like protein synthesis (9). Thus, previous studies have confirmed that CKS1B plays a vital role in the cancer cell growth, invasion, metastasis and chemical resistance. Nevertheless, deeper comprehension of the clinical prognosis and mechanistic explanation of CKS1B in pancreatic cancer is lacking.

As a novel therapeutic method, immunotherapy has become one of the research hotspots. PD-L1 (also known as B7-H1) is the main ligand of PD-1, mainly expressed in immune cells and tumor cells (10). Previous studies have shown that PD-L1 acts as an immune checkpoint to prevent the immune system from killing cancer cells by suppressing autoimmunity (11). Many studies have reported that high PD-L1 expression is closely related to poor survival in pancreatic cancer patients, which is an independent adverse prognostic factor (12–14). Therefore, tumor immunotherapy based on immune checkpoint blocker (ICB) has become the main method of tumor treatment nowadays (15). However, the efficacy of PD-1/PD-L1 blockers alone in the treatment of pancreatic cancer is actually poor, and only a few patients can benefit from immunotherapy currently (16, 17). Therefore, it is crucial to find effective biomarkers that can predict the efficacy of immunotherapy.

In this study, we investigated the expression and prognostic value of CKS1B in pancreatic cancer. Furthermore, the relationship between CKS1B and tumor immune microenvironment was also discussed. We hypothesized that knocking down CKS1B may suppress pancreatic cancer cell viability and migration by blocking PD‐L1 level. This study provides further insight into the function and detailed mechanism of CKS1B in pancreatic cancer and suggests that targeting CKS1B is a promising strategy for pancreatic cancer therapy.

## Method

### Pancreatic cancer dataset source and preprocessing

Public transcriptome and clinical data were acquired from The Cancer Genome Atlas (TCGA), Gene Expression Omnibus (GEO) and UCSC Xena Browser. RNA sequencing data (FPKM value) was retrieved for TCGA-PAAD, then converted into TPM values and log2 transformation was performed. We directly obtained the normalized matrix files for the GSE16515, GSE15471 and GSE62165 cohorts. Data on somatic mutation was also obtained from the TCGA database. The R language (version 4.1.2) was used to carry out all investigations.

### Pathway enrichment and Gene Set Enrichment Analysis (GSEA)

Co-expressed genes with CKS1B were defined by Pearson correlation analysis with correlation coefficient > 0.6 and p< 0.001. The protein-protein interactions (PPI) of CKS1B co-expressed genes were analyzed by STRING database (18) and visualized with Cytoscape software v3.9.1 (19). The Molecular Complex Detection (MCODE) plugin of the Cytoscape app was used to identify the densely connected regions/clusters in the PPI network (20). The top three gene clusters of the interactive network were extracted according to their scores. The “clusterprofiler” program was adopted for executing the gene ontology (GO) and Kyoto encyclopedia of genes and genomes (KEGG) pathway analyses (21). Premised on median levels of CKS1B expression, samples were further separated into two groups. We subsequently conducted the GSEA (22). The cut-off criteria for GSEA was p <0.05.

### Analysis of infiltration of immune cells

The corresponding infiltration status of 22 distinct immune cells in pancreatic cancer was assessed utilizing the ”CIBERSORT” software (23). Utilizing the ESTIMATE method, the stromal and immunological scores from each pancreatic cancer specimen were computed and analyzed.

### Patients

We obtained 25 paired tumors and normal tissues from the tissue bank of PLA General Hospital, which were collected between March and November 2018. The samples were diagnosed as pancreatic ductal adenocarcinoma by two pathologists. Additionally, the Ethics Committee of PLA General Hospital approved this research. All patients recruited into this study provided a formal informed consent form before participating.

### Tissue microarray and immunohistochemistry (IHC)

A pancreatic cancer tissue microarray (HPanA060CS02) that contained 37 cancer and 23 corresponding paracancerous tissues was acquired from Shanghai Outdo Biotech Company (Shanghai, China). IHC studies of CKS1B were performed on pancreatic cancer samples of tissue microarray. CKS1B antibody (DF3221, Affinity Biosciences) was used at a 1:500 dilution. IHC was performed according to the instructions. In brief, after dewaxing in xylene, rehydrating in alcohol, and blocking endogenous peroxidase activity, the tissue arrays were incubated overnight at 4°C with specific antibodies for CKS1B. After washing with PBS, the tissues were then incubated with a HRP-conjugated secondary antibody at 37°C for 40 min and 3,3’ -diaminobenzidine (DAB) for 5 min, then counterstained with hematoxylin for 30 s.

### Cell culture and transfection

ATCC (Manassas, VA, USA) supplied the human pancreatic ductal epithelium cell line HPDE6-C7 as well as pancreatic cancer cell lines Capan-1, BxPC-3, MIA PaCa-2, SW1990, and PANC-1. We then cultured cells in either RPMI 1640 or DMEM supplemented with 10% fetal bovine serum (FBS), and placed them in a 37°C, 5% CO2 incubator. After reaching 30% confluence, transfection of SW1990 cell was done utilizing shCKS1B or shNC (designed and synthesized by Jintuosi (Wuhan) Biotechnology Co., Ltd) using the Lipofectamine 3000 (L3000015, Invitrogen) following the manufacturer. CKS1B shRNA target sequences were: shRNA1: 5′-GGTCCATTATATGATCCAT-3′; shRNA2: 5′-GATGGGTCCATTATATGAT-3′.

### Reverse transcription-quantitative polymerase chain reaction (qRT‐PCR)

We undertook qRT-PCR to detect gene expression. Specifically, we isolated total RNA from cancer and normal samples utilizing TRIzol reagent (15596018, Ambion) and then converted it into cDNA using Eppendorf Mastercycler^®^. StepOnePlus Real-Time PCR System was utilized to execute qPCR premised on the primers listed in **Table 1**.

**Table 1:** Primers used for qRT‐PCR analysis.

Gene	Direction	Sequences (5′–3′)
18s	Forward	AACCCGTTGAACCCCATT
18s	Reverse	CCATCCAATCGGTAGTAGCG
CKS1B	Forward	TATTCGGACAAATACGACGACG
CKS1B	Reverse	CGCCAAGATTCCTCCATTCAGA
PD-L1	Forward	GGCATTTGCTGAACGCATTT
PD-L1	Reverse	ACAATTAGTGCAGCCAGGTCT

### Western blot assay

After BCA protein quantification, protein samples were transferred onto the PVDF membrane by SDS-PAGE. Following the blocking of the membranes using 5% non-fat milk, a primary antibody against CKS1B (DF3221, Affinity Biosciences), PD-L1 (13684, Cell Signaling Technology), LC3B (83506, Cell Signaling Technology), phosphorylated STAT3 (9145, Cell Signaling Technology) and GAPDH (97166, Cell Signaling Technology) was utilized to incubate the membranes throughout the night at 4°C, followed by incubation with corresponding peroxidase-labeled secondary antibodies. Based on the electrochemical luminescence (ECL) color development kit, protein levels were determined using a chemiluminescence detection system.

### CCK8 assay

Cell counting kit-8 (CCK-8) (E-CK-A362, Elabscience Biotechnology) was utilized to evaluate the proliferative capacity of cells. Cells were seeded into a 96-well plate, then 10 μL CCK-8 reagent was introduced into each well at different timepoint of 24 hours, 48 hours, 72 hours and incubated for 2 hours at 37°C. Following this, the absorbance at 450 nm was determined. The average OD values of three wells were calculated, and three repeated experiments were performed.

### Clone formation experiment

Following the use of trypsin to digest the cells, they were resuspended and counted. Subsequently, the cell suspension was plated in a six-well plate with 2000 cells/well. This was followed by additional incubation of the cells in an incubator comprising 5% CO2 at 37°C. After a total of 7 days, we fixed the cells with methanol for 30 min before staining them with Giemsa for 20 min. Finally, rinse using tap water and photograph for counting.

### Wound healing assay

Logarithmic growth cells were grown in a six-well plate with each well containing 5×10^5^ cells. Once the cells had attached to the six-well plate in a single layer, the six-well plate was scratched vertically using a 200μl pipette tip. After cleaning and removing the suspension cells with PBS, incubation was then carried out with serum-free medium (SFM) at 37°C with 5% CO2. Three separate replications of the experiment were carried out, and images were taken using a microscope at 0 and 24 hours after the experiment began.

### Transwell assay

After dilution to 2×10^5^/ml with SFM, 200 μl cell suspension was introduced into the upper chamber, while 600 μl medium that contained 20% FBS was introduced into the bottom chamber. A cotton tip was used to remove the upper surface of the membrane, and Giemsa stain was introduced into the lower surface following 24 hours of incubation at 37°C. The Matrigel matrix (356234, Corning) was diluted to 200 μg/mL with SFM, and then each Transwell^®^ insert was carefully filled with 100 μL of the diluted Matrigel matrix for invasion assays.

### Statistical analysis

The Wilcoxon matched-pairs signed-rank test was utilized to evaluate the differences in CKS1B mRNA levels that existed between malignant and corresponding paracancerous samples. The analysis was executed utilizing GraphPad Prism 8.4.3, and the data were presented as mean ± standard deviation. Unless otherwise noted, all experiments were performed at least thrice. Univariate Cox regression and multivariate Cox regression analyses were employed in order to evaluate significant factors that contributed to an independent prognosisusing the “survival” R package. When P-value is less than 0.05, statistical significance is regarded to have been achieved.

## Result

### CKS1B is highly expressed and correlated with histological grade in pancreatic cancer

**Figure 1:**
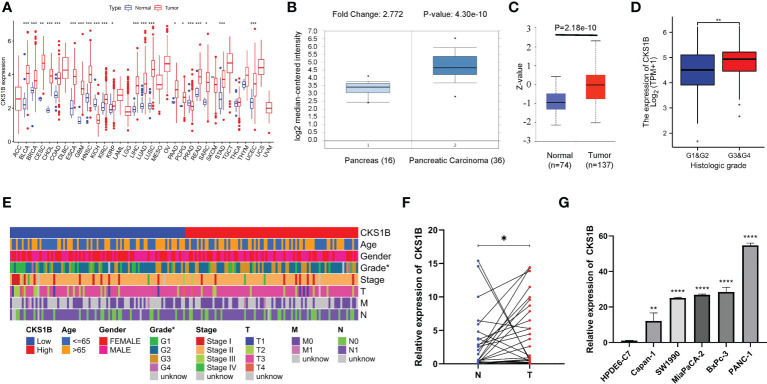
CKS1B is highly expressed and correlated with histological grade in pancreatic cancer. **(A)** CKS1B expression in different cancer types from TCGA datasets. **(B)** The mRNA expression of CKS1B in pancreatic cancer tissues in the Oncomine database. **(C)** The protein level of CKS1B in pancreatic cancer analyzed by the CPTAC database. **(D)** The clinical characteristics difference between high CKS1B and low CKS1B group. **(E)** The expression of CKS1B in different histological grade. **(F)** mRNA expression of CKS1B in pancreatic cancer tissues. **(G)** mRNA expression of CKS1B in cell lines. (*P < 0.05, **P < 0.01, ***P < 0.001, ****P < 0.001).

Based on information obtained from the TCGA database, the gene expression levels of CKS1B in various human cancers were compared with those found in normal tissues. A considerably elevated expression level of CKS1B mRNA was found in cancer tissues, including pancreatic cancer tissues (**Figure 1A**). The level of CKS1B expression was further validated using the Oncomine and GEO database, these findings illustrated that CKS1B was overexpressed in pancreatic cancer (**Figure 1B**; **Supplementary Figures 1A–C**). In addition, the findings from Clinical Proteomic Tumor Analysis Consortium (CPTAC) illustrated that the levels of CKS1B protein expression were elevated in pancreatic cancer contrast with normal samples (**Figure 1C**), revealing that the mRNA and protein expressions of CKS1B were comparable across multiple databases. Premised on the mean level of CKS1B expression, the patients were classified into low- and high CKS1B expression groups (**Table 2**), following which the relationship of CKS1B expression with clinical parameters was examined. CKS1B levels were substantially greater in G3-4 patients than that in G1-2 patients (**Figure 1D**). Additionally, high CKS1B expression was linked to higher histological grade in contrast with the low CKS1B expression (**Figure 1E**). Furthermore, we demonstrated differential expression of CKS1B in pancreatic cancer cells and tissues using qRT-PCR. According to the results, the CKS1B expression level was remarkably elevated in pancreatic cancer tissues (**Figure 1F**). Besides, we further determined the CKS1B expression in pancreatic cancer cell lines. Result illustrated that the CKS1B mRNA expression remarkably upregulated in pancreatic cancer cell lines in comparison to HPDE6-C7 (**Figure 1G**). The above results demonstrate that CKS1B is abnormally expressed in pancreatic cancer. In addition, IHC of tissue microarray also showed that CKS1B protein expression is higher in pancreatic cancer tissue (**Supplementary Figure 2**).

**Table 2:** The clinical characteristic between high CKS1B and low CKS1B group.

Characteristic	Low expression of CKS1B	High expression of CKS1B	p
n	89	89	
T stage, n (%)			0.145
T1	4 (2.3%)	3 (1.7%)	
T2	15 (8.5%)	9 (5.1%)	
T3	66 (37.5%)	76 (43.2%)	
T4	3 (1.7%)	0 (0%)	
N stage, n (%)			0.982
N0	24 (13.9%)	26 (15%)	
N1	61 (35.3%)	62 (35.8%)	
M stage, n (%)			1.000
M0	38 (45.2%)	41 (48.8%)	
M1	2 (2.4%)	3 (3.6%)	
Pathologic stage, n (%)			0.208
Stage I	13 (7.4%)	8 (4.6%)	
Stage II	69 (39.4%)	77 (44%)	
Stage III	3 (1.7%)	0 (0%)	
Stage IV	2 (1.1%)	3 (1.7%)	
Radiation therapy, n (%)			0.316
No	56 (34.4%)	62 (38%)	
Yes	26 (16%)	19 (11.7%)	
Gender, n (%)			0.292
Female	44 (24.7%)	36 (20.2%)	
Male	45 (25.3%)	53 (29.8%)	
Age, n (%)			0.764
<=65	48 (27%)	45 (25.3%)	
>65	41 (23%)	44 (24.7%)	
Residual tumor, n (%)			0.957
R0	55 (33.5%)	52 (31.7%)	
R1	26 (15.9%)	26 (15.9%)	
R2	2 (1.2%)	3 (1.8%)	
Histologic grade, n (%)			0.006
G1	22 (12.5%)	9 (5.1%)	
G2	49 (27.8%)	46 (26.1%)	
G3	16 (9.1%)	32 (18.2%)	
G4	1 (0.6%)	1 (0.6%)	
Alcohol history, n (%)			0.658
No	34 (20.5%)	31 (18.7%)	
Yes	48 (28.9%)	53 (31.9%)	
History of diabetes, n (%)			0.215
No	48 (32.9%)	60 (41.1%)	
Yes	22 (15.1%)	16 (11%)	
History of chronic pancreatitis, n (%)			0.693
No	62 (44%)	66 (46.8%)	
Yes	5 (3.5%)	8 (5.7%)	
Family history of cancer, n (%)			0.700
No	22 (20%)	25 (22.7%)	
Yes	33 (30%)	30 (27.3%)	
Age, mean ± SD	64.16 ± 11.08	65.34 ± 10.54	0.468

### CKS1B indicates a dismal prognosis in pancreatic cancer

In the pan-cancer dataset, an investigation of the connection between the CKS1B expression and patients’ prognoses was carried out. OS and PFS were included as survival metrics. According to Cox regression analysis of 33 types of cancer, CKS1B expression was significantly associated with OS in 13 types of cancers, including UVM, DLBC, UCEC, THCA, PCPG, PAAD, MESO, LUAD, LIHC, LGG, KIRP, KICH, and ACC (**Supplementary Figure 3A**). Besides, we investigated the possible link between the CKS1B expression and PFS in pancreatic cancer patients. The CKS1B expression affected PFS in 8 kinds of cancers, including ACC, HNSC, UVM, PAAD, LGG, PRAD, KIRP, UCEC, LIHC, and KICH (**Supplementary Figure 3B**). Kaplan-Meier survival curves illustrated that upregulated CKS1B expression was remarkably linked to unfavorable PFS and OS in patients with PAAD (**Supplementary Figures 3C, D**). To assess the diagnostic significance of CKS1B, we additionally generated a receiver operating characteristic (ROC) curve for further analysis. The value of the area under the curve (AUC) for CKS1B levels was 0.988 (CI = 0.978-0.999), indicating a strong potential for diagnostic application (**Supplementary Figure 3E**).

### CKS1B expression has independent prognostic value in pancreatic cancer

**Figure 2:**
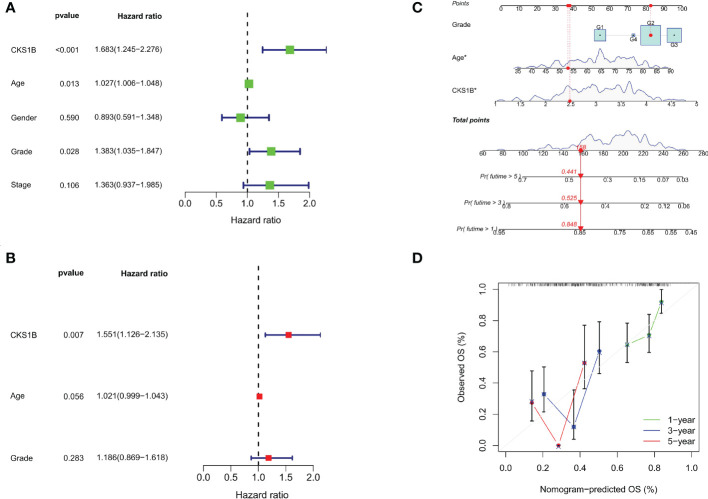
The independent prognostic analysis and nomogram construction in pancreatic cancer. **(A)** Univariate Cox regression analysis for CKS1B and clinical characteristics. **(B)**. Multivariate Cox regression analysis for CKS1B and clinical features. **(C)** The nomogram consists of age, grade, and CKS1B expression. **(D)** The calibration curve for evaluating model accuracy. (*P < 0.05).

A strong connection between high CKS1B expression and poor overall survival was discovered utilizing univariate Cox regression analysis (HR = 1.683, 95% CI = 1.245-2.276, P < 0.001) (**Figure 2A**). Moreover, multivariable regression analysis further supported that CKS1B independently served as a prognostic indicator in pancreatic cancer patients (HR = 1.554, 95% CI = 1.122-2.152, P = 0.008) (**Figure 2B**). And the test of Schoenfeld residuals indicate that the assumption of proportional hazards was not violated (p=0.3229, **Supplementary Figure 4**). Next, we developed a nomogram using age, grade, and CKS1B expression levels to anticipate 1-, 3-, and 5-year survival in pancreatic cancer patients (**Figure 2C**). In terms of the calibration curve, the 1-, 3-, and 5-year clinical outcomes were accurately predicted by the nomogram (**Figure 2D**). Together, these data indicate that CKS1B serves as an important biomarker for pancreatic cancer patients in predicting their overall survival.

### CKS1B is involved in immune and autophagy regulation through GSEA analysis

**Figure 3:**
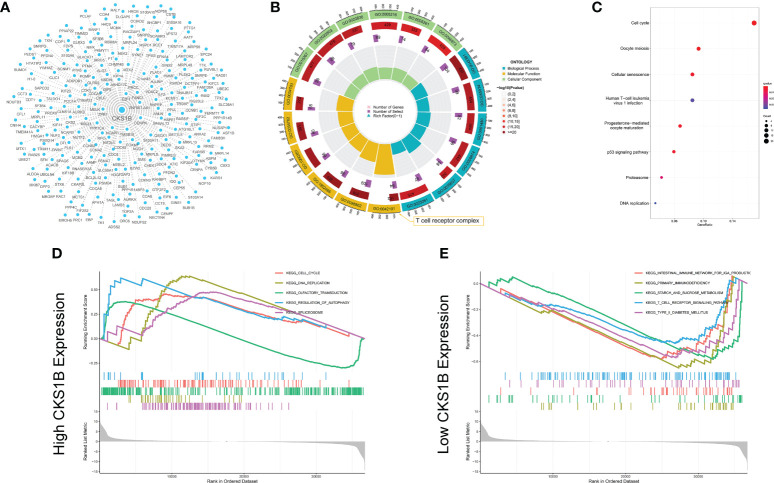
Co-expression network construction and enrichment analysis for CKS1B. **(A)** The co-expression network for 239 CKS1B co-expressed genes. **(B)** GO enrichment analysis for CKS1B based on CKS1B co-expressed genes. **(C)** KEGG pathway analysis for CKS1B based on CKS1B co-expressed genes. **(D)** GSEA enrichment analysis in high CKS1B group. **(E)** GSEA enrichment analysis in low CKS1B group.

We undertook KEGG pathway analysis and GSEA to determine the possible cellular processes that CKS1B involved in pancreatic cancer. Pearson correlation analysis was conducted to define CKS1B co-expressed genes. And 239 co-expressed genes were visualized by Cystoscope (**Figure 3A**), and the above genes were used for further enrichment analysis. CKS1B was enriched in the T cell receptor complex according to the result of GO enrichment analysis (**Figure 3B**). KEGG pathway analysis showed that CKS1B was related to the cell cycle, cellular senescence, DNA replication, p53 signaling pathway, etc. (**Figure 3C**). Furthermore, we analyzed the interaction of proteins corresponding to 239 CKS1B co-expressed genes using STRING database. The PPI network was performed by Cystoscope (**Supplementary Figure 5A**). The top three MCODE-generated clusters were exhibited in **Supplementary Figures 5B–D** based on the vertex weighting of the MCODE algorithm. Then KEGG enrichment analysis was also performed for genes in cluster 1, which was generally consistent with the analysis for all co-expressed genes (**Supplementary Figure 5E**). Besides, GSEA enrichment analysis also highlighted a substantial enrichment of autophagy regulation in the CKS1B high expression phenotype (**Figure 3D**), whereas the CKS1B low expression phenotype underwent a substantial enrichment in the T-cell receptor signaling pathway (**Figure 3E**).

### CKS1B expression is related to the infiltration of immune cells in pancreatic cancer tissue

**Figure 4:**
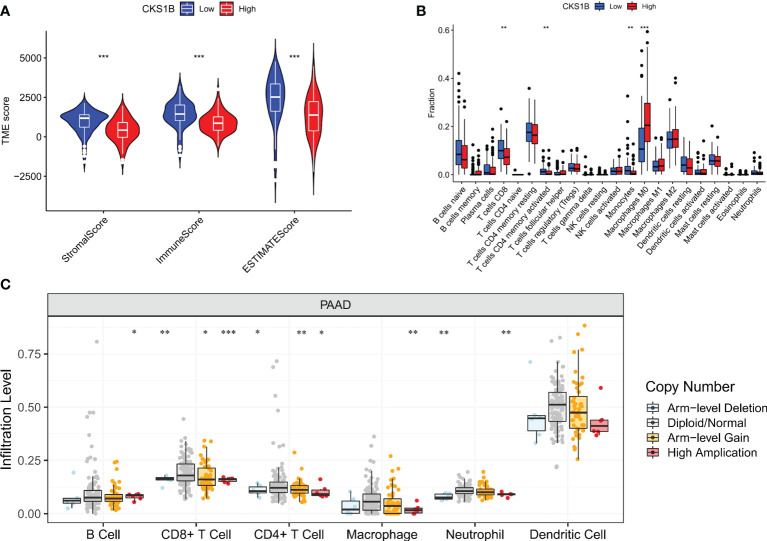
CKS1B expression is associated with immune cell infiltration in pancreatic cancer. **(A)** The immuneScore, stromalScore, and ESTIMATEScore between high and low CKS1B group. **(B)** The level of immune cell infiltration between the groups with high and low CKS1B expression. **(C)** The correlation of CKS1B somatic copy number alterations with immune cell infiltration. (*P<0.05,**P<0.01,***P<0.001).

**Figure 5:**
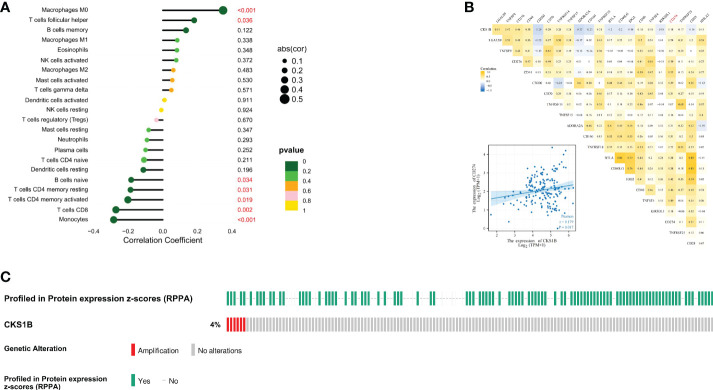
The expression of CKS1B correlates with immune checkpoint in pancreatic cancer. **(A)** The correlation of CKS1B expression with immune cells. **(B)** The relationship between CKS1B expression and immune checkpoints. **(C)** The genetic alteration of CKS1B.

Afterward, we compared the immuneScore and the infiltration levels of immune cells between CKS1B high and low expression groups to examine the involvement of CKS1B in the pancreatic cancer immune microenvironment. The average immuneScore, stromalScore, and ESTIMATEScore were higher in the low CKS1B expression group (**Figure 4A**). And the abundance of infiltration of 22 different kinds of immune cells is displayed (**Figure 4B**). The high-CKS1B expression group had a higher abundance of M0 macrophages, and a lower abundance of CD4 memory-activated T cells, CD8 T cells, and monocytes than that low-CKS1B expression group. Our study analyzed the relationship between CKS1B somatic copy number alterations and immune cell infiltration in pancreatic cancer samples using TIMER. Our data indicated that CKS1B somatic copy number alterations were substantially linked to the infiltration degree of neutrophils, CD4+ T cells, macrophages, B cells, and CD8+ T cells (**Figure 4C**). Additionally, infiltration levels were highest in diploid/normal samples. Furthermore, immunocell correlation analysis illustrated a positive link between CKS1B and M0 macrophages and follicular helper T cells, but an inverse link to naive B cells, CD4 memory-resting T cells, CD4 memory-activated T cells, CD8 T cells, and monocytes (**Figure 5A**). In addition, an investigation of the connection between the expression of CKS1B and immune checkpoints was carried out. Furthermore, we discovered a positive link between PD-L1 expression and CKS1B levels (P=0.017) (**Figure 5B**), which was also verified in GSE16515, GSE15471 and GSE62165 cohorts (**Supplementary Figures 6A–C**). Subsequently, the genetic alteration in CKS1B was studied in 149 pancreatic cancer samples (TCGA, Firehose Legacy). It was found that the CKS1B genetic alteration occurred 4% across 149 pancreatic cancer patients (**Figure 5C**). The type of mutation most commonly occurred among them was amplification. Additionally, the reverse-phase protein arrays (RPPA) identified the CKS1B protein was frequently expressed in pancreatic cancer tissues.

### CKS1B expression is correlated with microsatellite instability (MSI), tumor mutational burden (TMB), and neoantigen

**Figure 6:**
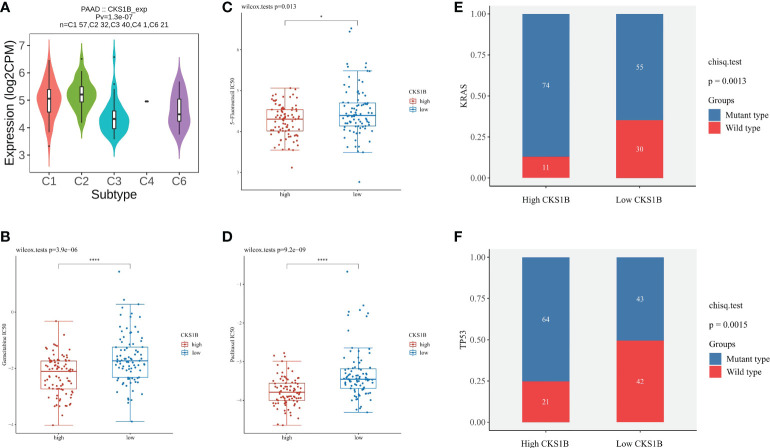
The chemotherapy sensitivity difference between high and low CKS1B group. **(A)** The relationship between CKS1B expression and pancreatic cancer immune subtypes. **(B–D)** The IC50 of common chemotherapeutic agents in high CKS1B and low CKS1B patients (gemcitabine, 5-fluorouracil, and paclitaxel). **(E, F)** KRAS and TP53 mutation status in the high and low CKS1B groups. (*P < 0.05, ****P < 0.0001).

To gain a deeper comprehension of the function performed by CKS1B in the tumor microenvironment (TME), the link between CKS1B expression and TMB, MSI, and neoantigens was investigated. There is mounting evidence that neoantigens, MSI, and TMB in TME are linked to antitumor immunity, which may anticipate the effectiveness of tumor immunotherapy (24). According to our results, CKS1B expression exhibited significant positive correlations with TMB in STAD, LUAD, SARC, LGG, HNSC, LUSC, BRCA, PAAD, BLCA, and ACC, and negative relations in THYM (**Supplementary Figure 7A**). Positive links were discovered between MSI and CKS1B expression in HNSC, LIHC, SARC, BLCA, and STAD and negtive links in PRAD (**Supplementary Figure 7B**). We further observed a positive link between CKS1B expression and neoantigens in BLCA, BRCA, LUAD, and LUSC (**Supplementary Figure 7C**). It could be summarized that CKS1B was significantly positively linked to TMB in pancreatic cancer (R=0.49, P=1.4e-10) (**Supplementary Figure 7D**), which confirmed our hypothesis that CKS1B may exert anti-tumor immunity by influencing immune microenvironment. Next, with the TISIDB website, the role of CKS1B expression in pancreatic cancer subtypes was explored. It has been reported that immune types can be classified into six categories: wound healing (C1), IFN-γ dominant (C2), inflammatory (C3), lymphocyte depleted (C4), immunologically quiet (C5), and TGF-β dominant (C6) (25). These findings illustrated that the levels of CKS1B expression in distinct immune subtypes of PAAD were significantly different (**Figure 6A**). Additionally, CKS1B was discovered to be expressed at a high level in C2 subtypes and lowly expressed in C3 subtypes. According to the pRRophetic algorithm, three common chemotherapeutic agents (gemcitabine, 5-fluorouracil, and paclitaxel) were studied in high CKS1B and low CKS1B patients and found that these drugs had lower IC50 in high CKS1B patients (**Figures 6B–D**). It followed that these three drugs appeared to be more sensitive to patients with high levels of CKS1B. Additionally, KRAS and TP53 mutation status differed considerably in the low- and high-CKS1B groups (**Figures 6E, F**).

### CKS1B is positively associated with efficacy of immunotherapy in pancreatic cancer

**Figure 7:**
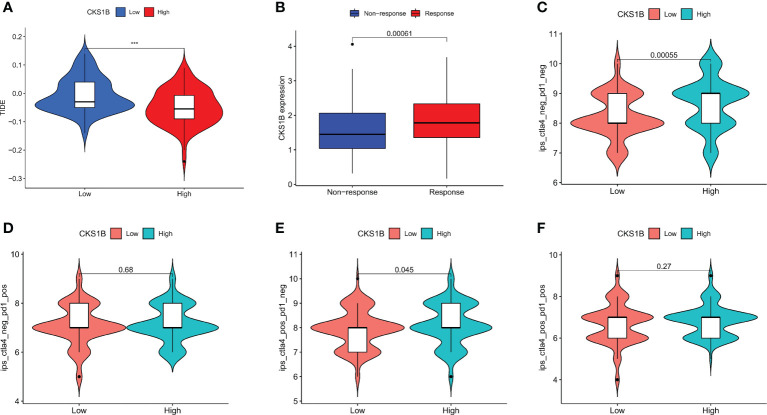
CKS1B is positively associated with efficacy of immunotherapy in pancreatic cancer. **(A)** The TIDE score in high CKS1B and low CKS1B groups. **(B)** The comparison of beneficiaries from immunotherapy between the high- and low-CKS1B group. **(C–F)** Correlation analysis between immunophenoscore of anti-CTLA-4 and anti-PD-1 blocker and CKS1B eexpression. (***P<0.001).

To better elucidate the value of CKS1B in predicting immunotherapy response, we analyzed the tumor immune dysfunction and exclusion (TIDE) score in PAAD patients of TCGA. The results showed that the TIDE score was lower in the high CKS1B group (**Figure 7A**). In addition, the IMvigor210 cohort including 348 urothelial carcinoma patients who received immunotherapy were enrolled for analysis. We found that the responder group was positively associated with CKS1B expression, indicating that patients in the high CKS1B group had a better response to immunotherapy (**Figure 7B**). The immunological score could predict the anti-CTLA-4 and anti-PD-1 antibody response, which can identify determinants of tumor immunogenicity. Subsequently, we investigated this correlation of immunophenoscore in the TCGA-PAAD cohort and found that risk groups in IPS- PD1 and IPS-PD1-CTLA4 blocker scores had no significant difference in immunophenoscore. Whileas, IPS and IPS-CTLA4 were higher in the high-risk group suggesting better immunotherapeutic benefits (**Figures 7C–F**).

### Knocking down CKS1B inhibits autophagy and STAT3/PD-L1 signaling in PC cells

**Figure 8:**
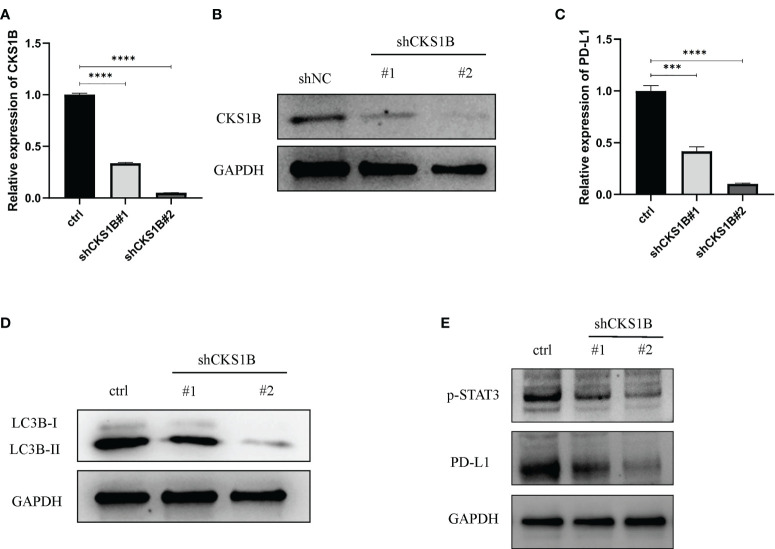
Knocking down CKS1B inhibits autophagy and STAT3/PD-L1 signaling in PC cells. **(A, B)** mRNA and protein expression of CKS1B in cells transfected with shCKS1B. **(C)** mRNA expression of PD-L1 was measured by qRT-PCR. **(D)** Protein levels of LC3B were measured by Western blotting. **(E)** Protein levels of p-STAT3 and PD-L1 were measured by western blotting. (***P<0.001,****P<0.0001).

To attain more insights into the function performed by CKS1B in the progression of pancratic cancer, CKS1B was silenced in SW1990 cells by transfecting them with shCKS1B plasmids (**Figures 8A, B**). As mentioned above, we found that CKS1B was involved in the regulation of autophagy by GSEA analysis, so we further detected whether the autophagy level of pancreatic cancer cells changed after CKS1B knockdown by western blotting. LC3B, which is a widely used marker of autophagy, was investigated in the presence of CKS1B depletion in our research. The result showed that CKS1B knockdown cells had significant reductions in LC3-II (**Figure 8D**), which demonstrated that CKS1B was associated with autophagic activity in pancreatic cancer. In view of the important role of CKS1B in pancreatic cancer immunotherapy, we further explored the association between CKS1B and PD-L1 and its possible regulatory mechanism in pancreatic cancer cell lines. First, qRT-PCR analysis was utilized to evaluate the PD-L1 expression after CKS1B knockdown, and the findings illustrated that PD-L1 expression was attenuated substantially following CKS1B depletion (**Figure 8C**). Subsequently, PD-L1 and p-STAT3 expression were measured in SW1990 cells with suppressed CKS1B by western blotting. It was observed that CKS1B knockdown remarkably lowered the levels of PD-L1 and p-STAT3 proteins (**Figure 8E**), implying that CKS1B affects PC cell function by modulating STAT3/PD-L1 signaling.

### CKS1B knockdown inhibits the proliferation of pancreatic cancer cells

**Figure 9:**
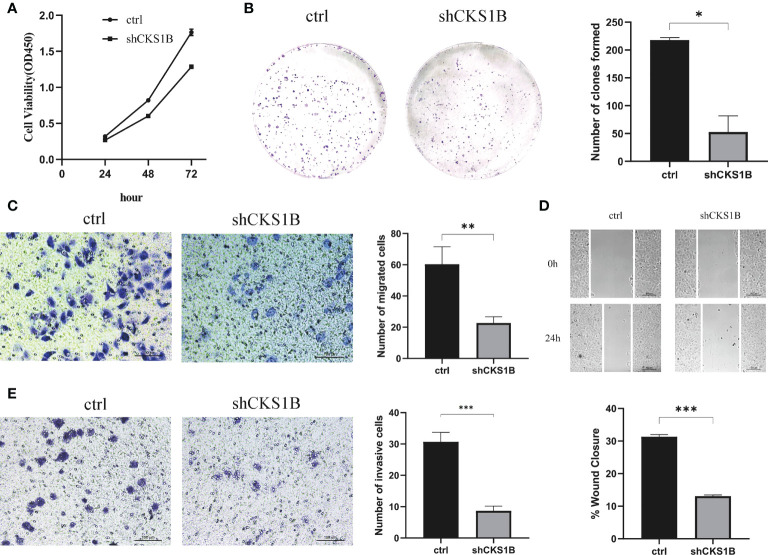
CKS1B promotes cell proliferation, migration and invasion in pancreatic cancer. **(A, B)** Cell viability of different groups of cells was measured by CCK8 assay **(A)** and colony formation assay **(B)**. **(C, D)** Cell migration ability of different groups of cells was detected by transwell assay **(C)** and wound healing assays **(D)**. **(E)** Cell invasion ability of different groups of cells was detected by transwell assay. (*P<0.05,**P<0.01,***P<0.001).

According to the above qRT-PCR result, we found that knockdown efficiency was 66.5% for cells treated with shCKS1B#1 and 95.1% for cells treated with shCKS1B#2. Therefore, we selected the group with higher knockdown efficiency for subsequent experiments. Upon successful shCKS1B transfection, the CCK8 assay demonstrated that cell viability was drastically reduced (**Figure 9A**). Our results showed that the blocking of CKS1B could significantly reduce colony formation compared with the control group (**Figure 9B**). These findings provided evidence that CKS1B is responsible for promoting the proliferative potential of pancratic cancer cells.

### Knockdown of CKS1B inhibits migratory and invasive capabilities of pancreatic cancer cells

Transwell migration assays and wound healing assays were performed to assess the effect of CKS1B on the migratory capacity of pancreatic cancer cells. The results showed that the migration rate of SW1990 cells transfected with shCKS1B was significantly lower than that of control cells (**Figure 9C**). And wound healing assays illustrated that the migratory rate of SW1990 cells with shCKS1B transfection was 13.09 ± 0.37%, lower than that control group 31.32 ± 0.66%, which implied that CKS1B enhanced the migratory capacity of pancreatic cancer cells (**Figure 9D**). As demonstrated by transwell invasion assays, the invasiveness of SW1990 cells infected with shCKS1B was also reduced considerably in contrast with the control group (**Figure 9E**).

## Discussion

Pancreatic cancer is a kind of malignant neoplasm that leads to a poor prognosis and present no sign in its early stages (26). By the year 2030, it is anticipated that pancreatic cancer will become the second major contributor to cancer-associated fatalities in the United States (27). Despite many advances in pancreatic cancer research in recent years, the 5-year survival rate remains below 10% (28). Thus, there is an urgent need to find new therapeutic approaches to improve the long-term survival of patients with pancreatic cancer. Immunotherapy, represented by immune checkpoint inhibitors, as a new antitumor therapy, has shown good therapeutic effect in non-small cell lung cancer, melanoma, lymphoma and other malignant tumors (29–31). However, clinical data showed that single immune checkpoint inhibitors have limited therapeutic effect on pancreatic cancer, which may be related to its unique tumor microenvironment (32–34). Therefore, it is particularly important to find markers that could predict the effect of immunotherapy for pancreatic cancer.

Several researches have illustrated that CKS1B performs a function in cancer progression. It was reported that CKS1B expression could be suppressed by miR-1258, inhibiting colorectal cancer proliferation and migration (35). Besides, silencing CKS1B could limit the capacity of retinoblastoma (RB) cells proliferation, and migration, as well as angiogenesis by inhibiting MEK/ERK activation (36). In addition, CKS1B is considered a predictor of adverse survival in patients with multiple myeloma (37). Furthermore, CKS1B has been reported to be a resistance-inducing gene. At present, the research on CKS1B targeted therapy is also in full swing. Studies have shown that miR-204 can down-regulate the expression of CKS1B in gastric cancer (38). Other experiments showed that CKS1B expression was positively regulated by MALAT1, which provided a new adjunct strategy for improving the efficacy of radiotherapy in ESCC (39). Additionally, it was also found that 3-O-(Z)-coumaroyloleanolic acid, a candidate compound for targeting CKS1B, can reverse CKS1B-induced chemoresistance in lung cancer (40). Consistent with previous studies, we found that CKS1B was highly expressed in multiple cancers including pancreatic cancer, which was confirmed in the Oncomine and CPTAC databases. Besides, qRT-PCR also substantiated that CKS1B was upregulated in pancreatic cancer cells and tissues. Furthermore, CKS1B was substantially linked to histological grading in pancreatic cancer. Additional research was conducted to investigate the predictive performance of CKS1B. Results from the Kaplan-Meier survival analysis were consistent with those of the univariate Cox analysis, which illustrated that CKS1B expression is substantially linked to OS and PFS in pancreatic cancer. Furthermore, as an independent prognostic factor, CKS1B was incorporated into a nomogram that can accurately anticipate patients’ OS over 1, 3, and 5 years.

Tumor immune microenvironment (TIME) has been proven to play a significant role in pancreatic cancer development (41, 42). In pancreatic cancer immune microenvironment, anti-tumor immune cells such as CD4^+^/CD8^+^ T cells, NK cells and DCs are less, while immune-suppressive cells such as Tregs, MDSCs and TAMs are abundant. The immunosuppressive tumor microenvironment in pancreatic cancer suppresses the anti-tumor immune response and cause immune escape, thus affecting the effect of immunotherapy for pancreatic cancer (43). In our research, we observed that the T cell receptor signaling pathway was substantially enriched in the low-CKS1B group, which showed that CKS1B may contribute to immune regulation. In addition, we also found that immuneScore, stromalScore, and ESTIMATEScore were lower in the high-CKS1B expression group, which indicated that high-CKS1B expression group may be under an immunosuppressed state. Studies have shown that tumor-infiltrating immune cells is closely related to tumor progression and prognosis (44). Additionally, CD4^+^ T cells have synergistic effects with cytotoxic CD8+ T cells, which could activate antitumor immune responses (45, 46). It was also found that high levels of M2 macrophages and Treg cells in tumor-infiltrating cells were significantly associated with poorer survival, while high levels of CD4^+^ T, CD8^+^ T and M1 macrophages were significantly associated with higher survival rate in pancreatic cancer patients (47, 48). In our study, we found that high-CKS1B expression group had a higher abundance of M0 macrophages, and a decreased abundance of CD4 memory-activated T cells, monocytes, and CD8 T cells compared to the low CKS1B expression group. These results indicate that the tumor microenvironment with high CKS1B expression exhibited highly immunosuppressive characteristic, which provided a microenvironment condition for CKS1B to promote tumor development, invasion and metastasis. And the two interact to jointly maintain the malignant progression of pancreatic cancer. In addition, the relationships between the CKS1B expression and immune checkpoints were further investigated. We discovered a positive link between PD-L1 expression and CKS1B levels. What’s more, we evaluated the connections between CKS1B expression and neoantigens, MSI, and TMB, which may anticipate the treatment effectiveness of tumor immunotherapy. The result illustrated that CKS1B was significantly positively linked to TMB in PC. The above results showed high CKS1B patients were much more likely to benefit from anti-PD-L1 treatment.

Recently, targeted autophagy has emerged as a new approach to cancer therapy (49). Similarly, studies have shown that autophagy contributes to the onset and advancement of pancreatic cancer (50, 51). In addition, autophagy has also been associated with chemotherapy resistance of pancreatic cancer cells (52). In our research, further GSEA analysis showed that CKS1B was involved in the regulation of autophagy, which was further verified by western blotting. The result showed that LC3-II levels were remarkably lowered following the CKS1B knockdown. Furthermore, qRT-PCR analysis illustrated a considerable decrease in PD-L1 expression following the knockdown of CKS1B. However, it is unclear how CKS1B regulates PD-L1. Studies have shown that STAT3 is an upstream molecular of PD-L1 (53). Many studies have reported that CKS1B may modulate STAT3 signaling. Generally, the CKS1B/STAT3 axis contributes to the development of cancer. A previous research report conducted by Liu et al. illustrated that CKS1B contributes to HCC cell proliferation and metastasis by stimulating the JAK/STAT3 signaling (54). Another study highlighted that the drug resistance of myeloma cells may be induced through the stimulation of the STAT3 signaling by CKS1B (55). In addition, CKS1B/STAT3/PD-L1 axis has also been reported in several studies. A study by Wang et al. demonstrated that CKS1B overexpression may increase PTC viability and invasiveness by altering Akt phosphorylation and STAT3/PD-L1 signaling pathways (56). Another study also showed that PD-L1 expression may be enhanced by CKS1B/STAT3 axis, further promoting lung cancer development (57). In the present study, CKS1B was also observed to favorably modulate STAT3/PD-L1 signaling in PC cells, and blocking CKS1B might inhibit the development of PC *via* STAT3/PD-L1 signaling.

## Conclusion

In conclusion, we first used public databases to examine the differential expression and prognostic significance of CKS1B in pancreatic cancer. Then, further analysis showed that CKS1B was significantly associated with immune infiltration and could predict the immunotherapy effect for pancreatic cancer. Finally, we demonstrated that knocking down CKS1B may suppress PC cells’ viability and migratory capacities by suppressing autophagy and STAT3/PD-L1 signaling. These results offer a deeper knowledge of the function performed by CSK1B/STAT3/PD-L1 in PC advancement. Nevertheless, our study does have some limitations. Firstly, how CKS1B regulates autophagy remains unclear, although STAT3 has also been reported to be involved in autophagy. Secondly, it is not known if other signaling pathways play a role in this process. In addition, the function of CKS1B *in vivo* needs to be investigated in further study.

## Data availability statement

The original contributions presented in the study are included in the article/**Supplementary Material.** Further inquiries can be directed to the corresponding author.

## Ethics statement

The studies involving human participants were reviewed and approved by the Ethics Committee of PLA General Hospital. The patients/participants provided their written informed consent to participate in this study.

## Author contributions

LL, JW, and ZZ contributed equally to this work, and were considered as the co-first author. LL participated in design and conception of this study. JW performed part of the experiment. ZZ downloaded the data form corresponding database. QY and ZD was responsible for sample collection and processing. WZ and XG performed the bioinformatics analysis in software. KP and CL were responsible for experimental guidance. All author participated in writing the manuscript. RL proposed the conception and revised the manuscript. All authors read and approved the final manuscript.

## Acknowledgments

We acknowledge TCGA and GEO databases for providing their platforms and contributors for uploading their meaningful datasets.

## Conflict of interest

The authors declare that the research was conducted in the absence of any commercial or financial relationships that could be construed as a potential conflict of interest.

## Publisher’s note

All claims expressed in this article are solely those of the authors and do not necessarily represent those of their affiliated organizations, or those of the publisher, the editors and the reviewers. Any product that may be evaluated in this article, or claim that may be made by its manufacturer, is not guaranteed or endorsed by the publisher.

## Abbreviations

PC, Pancreatic cancer; CKS1B, CDC28 protein kinase regulatory subunit 1B; TCGA, the Cancer Genome Atlas; CPTAC, the Clinical Proteomic Tumor Analysis Consortium; STAT3, Signal transducer and activator of transcription 3; IHC, immunohistochemistry; GO, Gene ontology; KEGG, Kyoto encyclopedia of genes and genomes; TMB, Tumor mutation burden; MSI, Microsatellite instability; GSEA, Gene set enrichment analysis; TME, Tumor microenvironment; ROC, Receiver operating characteristic; TIDE, Tumor immune dysfunction and exclusion; TIME, Tumor immune microenvironment.
