# Supplementary material for: Identification of CKS1B as a prognostic indicator and a predictive marker for immunotherapy in pancreatic cancer
Source: Front Immunol. 2022 Nov 3;13:1052768. doi: 10.3389/fimmu.2022.1052768 (PMC9668883; doi:10.3389/fimmu.2022.1052768)
Supplement: Supplementary file 1 [file DataSheet_1.docx]

**
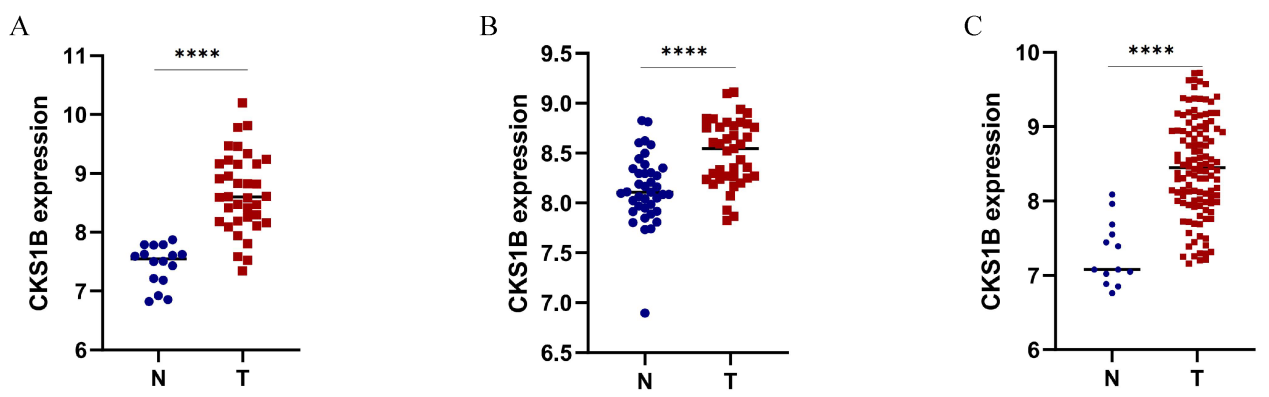
**

**Supplementary Fig.1** The expression of CKS1B in GSE16515 (A), GSE15471 (B) and GSE62165 (C) cohorts. (****P < 0.0001)


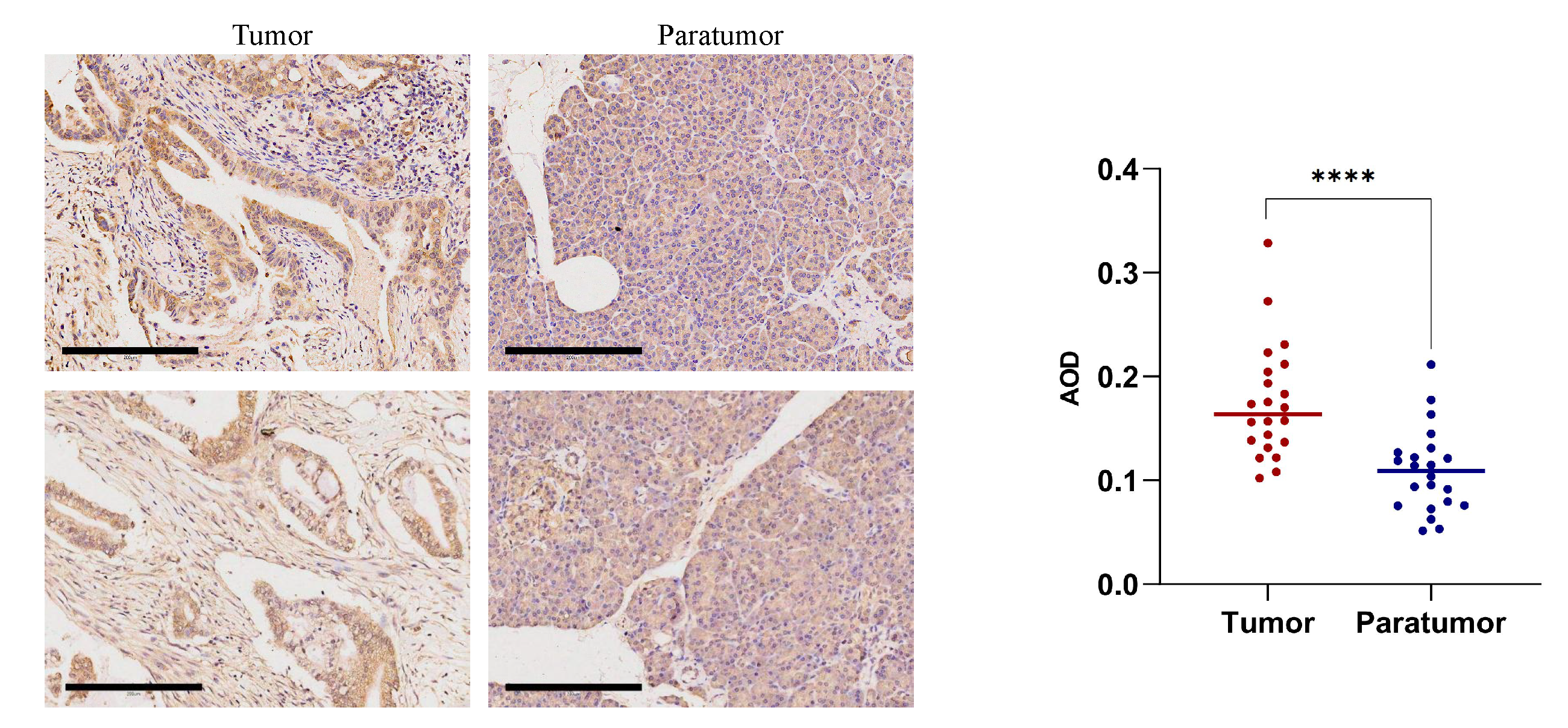


**Supplementary Fig. 2** IHC for 23 paired pancreatic cancer tissues and adjacent tissues. (****P < 0.0001)


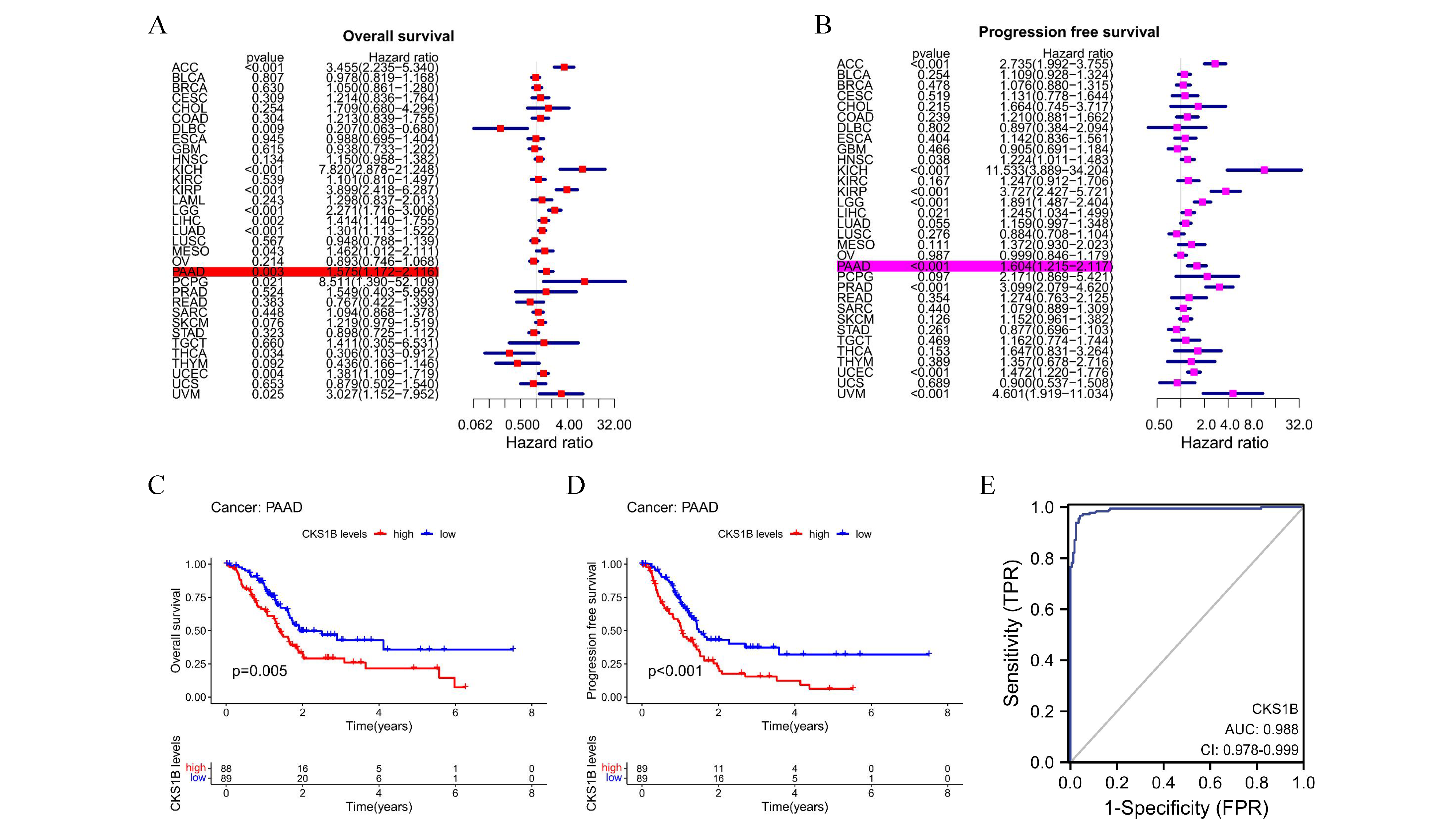


**Supplementary Fig. 3** Prognostic Value of CKS1B in pancreatic cancer. A. Forest plot of the association between CKS1B expression and OS in 33 types of tumor. B. Forest plot of the association between CKS1B expression and PFS in 33 types of tumor. C. Kaplan-Meier analysis of the association between CKS1B expression and OS. D. Kaplan-Meier analysis of the association between CKS1B expression and PFS. E. ROC curve to assess sensitivity and specificity of CKS1B expression as a diagnostic biomarker for pancreatic cancer in TCGA database.

**
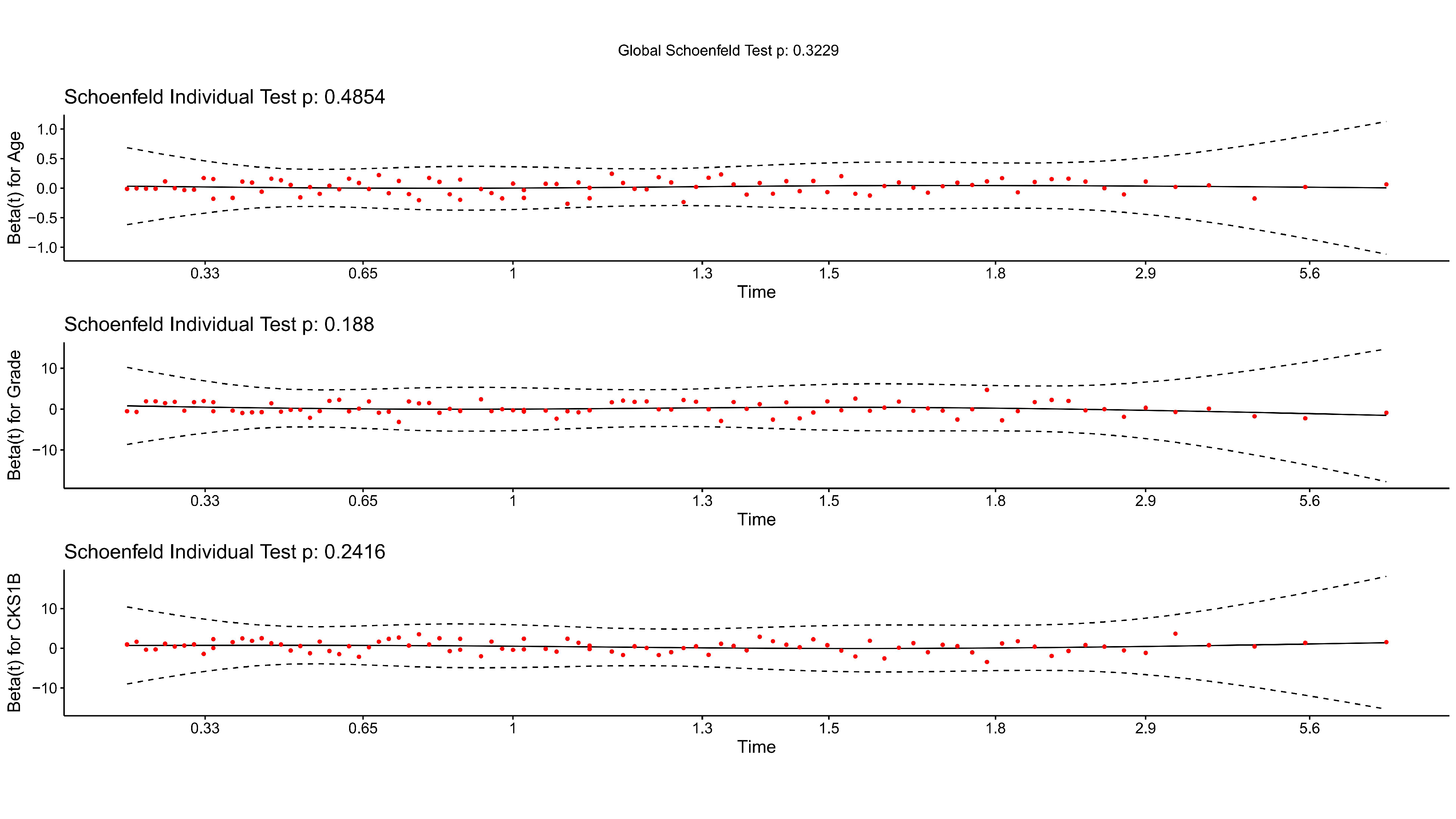
**

**Supplementary Fig. 4** The Schoenfeld residuals test to examine the CoxPH model assumptions.

**
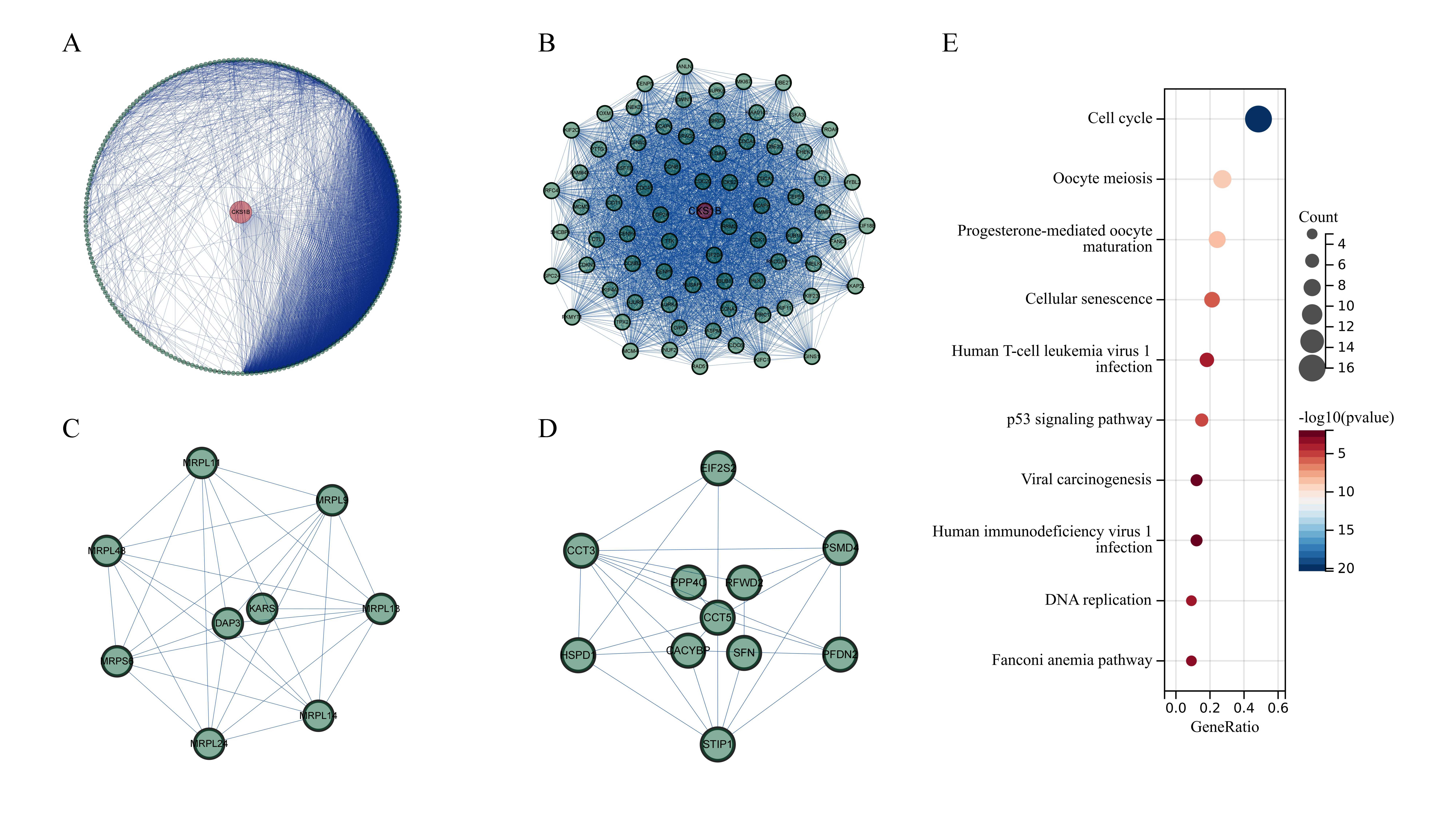
**

**Supplementary Fig. 5** The protein-protein interactions (PPI) network based on CKS1B co-expressed genes. A. The protein–protein interacting network was visualized by Cytoscape. B-D. The top three clusters derived from the protein-protein interactions network using MCODE. (B)-cluster 1, (C)-cluster 2, and (D)-cluster 3. E. KEGG pathway analysis for genes in cluster 1.

**
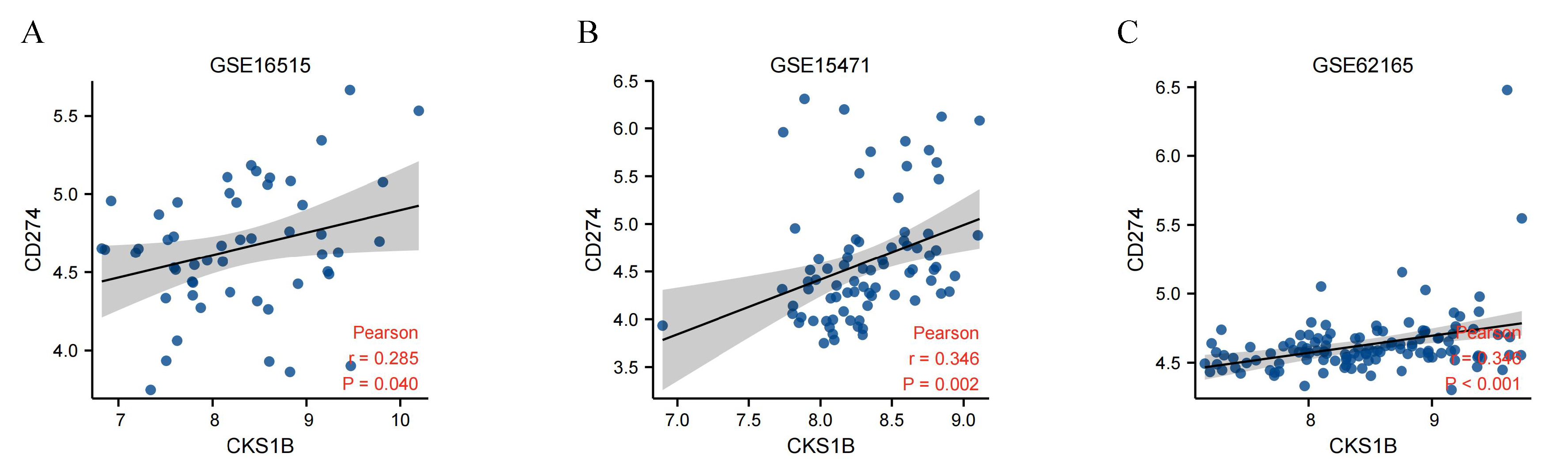
**

**Supplementary Fig. 6** The relationship between CKS1B expression and PD-L1 expression in GSE16515 (A), GSE15471 (B) and GSE62165 (C) cohorts.


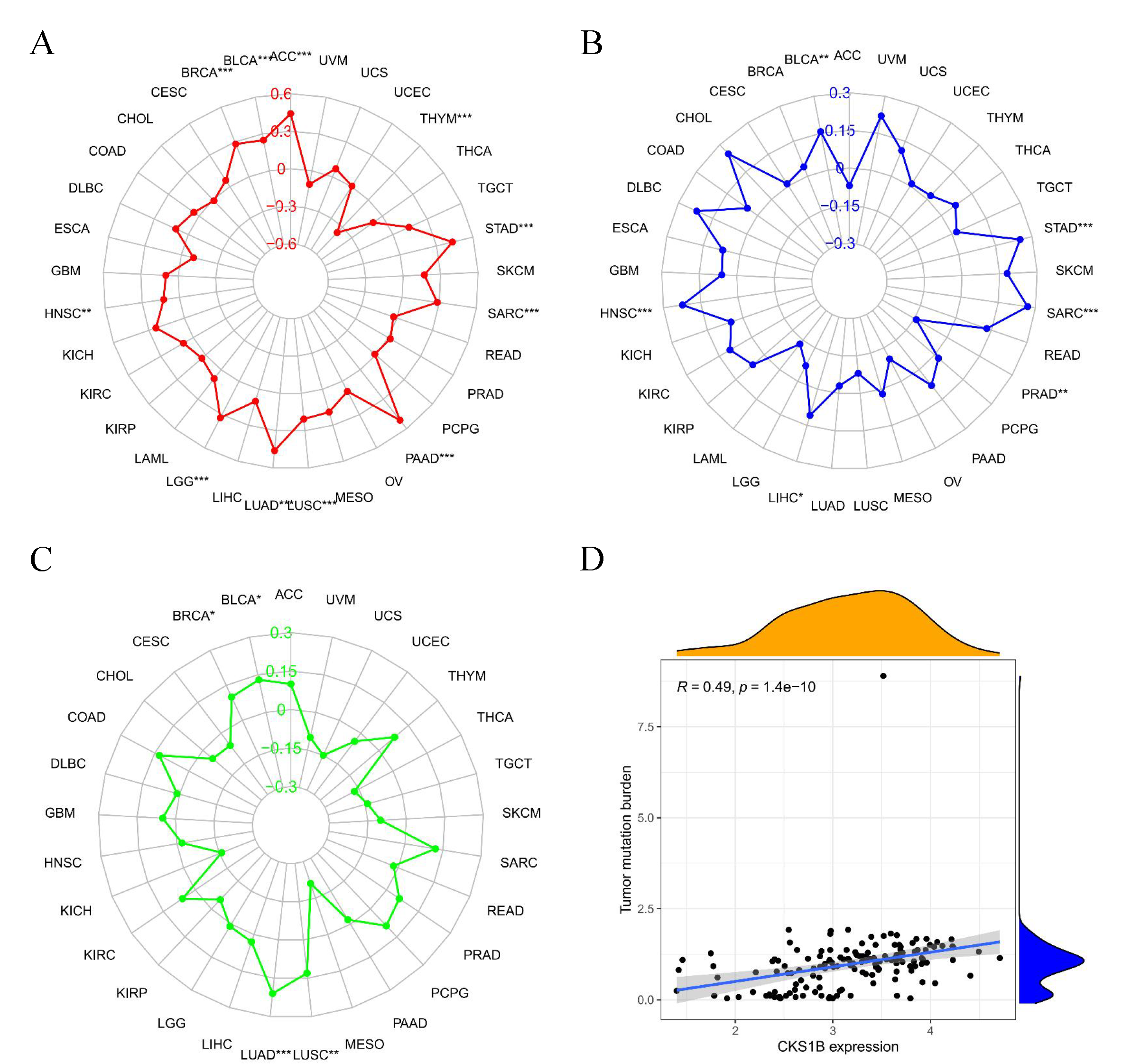


**Supplementary Fig. 7** The relationship between CKS1B and genome heterogeneity in pan-cancer. A-C. The relationship between CKS1B expression and TMB (A), MSI (B) and neoantigen (C) in human cancers. D. The correlation between CKS1B expression and TMB in pancreatic cancer.
